# Development of simulation education debriefing protocol with faculty guide for enhancement clinical reasoning

**DOI:** 10.1186/s12909-019-1633-8

**Published:** 2019-06-11

**Authors:** Juyeon Bae, JuHee Lee, Yeonsoo Jang, Yoonju Lee

**Affiliations:** 1 College of Nursing, Yonsei University, Seoul, Korea; 2 Mo-Im Kim Nursing Research Institute, College of Nursing, Yonsei University, 510 College of Nursing, Yonsei-ro 50, Seodaemun-gu, Seoul, 03722 Korea; 3 Mo-Im Kim Nursing Research Institute, College of Nursing, Yonsei University, 505 College of Nursing, Yonsei-ro 50, Seodaemun-gu, Seoul, 03722 Korea; 4 College of Nursing, Pusan National University, Gyeongsangnam-do, Korea

**Keywords:** Clinical reasoning, Debriefing, Undergraduate, Protocol, Simulation education

## Abstract

**Background:**

The clinical environments are more complex, diverse and rapidly changing. Patients’ conditions were chronic and complexed, healthcare providers require clinical reasoning for patient safety care. One of the learning methods to foster clinical reasoning is simulation education. The aim of this study was to develop a simulation education debriefing protocol that can improve clinical reasoning.

**Methods:**

This study was conducted following steps. For the first step, the literature review was performed to constitute a preliminary debriefing protocol. Secondly, content validity was evaluated by five simulation learning experts. Thirdly, in-depth interview was conducted to re-examine content validity with education experts. Finally, the final debriefing protocol was applied to 7 undergraduate nursing students to examine feasibility of the protocol.

**Results:**

The protocol consisted of debriefing steps, learning outcome, clinical reasoning attributes, core questions and guideline for instructor. Results of applicability of debriefing protocol represented that participants mentioned their reasoning competency was improved and understood the overall flow of reasoning.

**Conclusions:**

The debriefing protocol is important to educate healthcare providers ‘clinical reasoning. It would be able to contribute to develop healthcare providers’ clinical competencies.

**Electronic supplementary material:**

The online version of this article (10.1186/s12909-019-1633-8) contains supplementary material, which is available to authorized users.

## Background

The 4th Industrial Revolution leads to the development of technologies such as artificial intelligence. Artificial intelligence is smarter, thereby, routine procedures were replaced by the robots. Up-to-date knowledge, information were more easily assessed to healthcare providers. These trends led to changes in healthcare education [1]. As the core tasks, such as the skilled use of making judgments, are expected to correspond to human being, the nurse’s ability to conduct clinical reasoning clearly makes decisions in the performance of care and may improve the quality of care [2]. Clinical environments are more complex and diverse, patients’ health problems were complicated, accuracy action, wise judgment is connected to patient safety [3]. As such clinical environment changes, clinical reasoning is core competency for healthcare providers [4, 5]. Simmons [4] defines clinical reasoning as thinking strategies to examine and analyze the data relevant to patients, apply nursing processes to solve the patients’ problems, make nursing diagnoses, and create nursing plans accordingly. Thus, clinical reasoning is a thinking process of exploring experiences and systematizing thoughts. The process of reasonable conclusion occurs by collecting and analyzing data in a logical way via the process of discussion [6]. The clinical reasoning was multilayered and multiple elements competency for healthcare providers to make accurate decision in complex and uncertain patients’ conditions [3]. The high capacity of reasoning was linked with right decision-making and positive influence on patient safety by solving patients’ health problems [6].

As clinical reasoning ability is emphasized as a core competency of healthcare providers, the importance of education is increasing. American Association of Colleges of Nursing (AACN) [7] emphasized clinical reasoning for baccalaureate education, explained as a vitally required competency for integrative problem solving. Nursing education is provided with the application of nursing processes as a fundamental framework [8]. Previous studies stated clinical reasoning competence can occur through acquiring knowledge via lectures and repeated clinical practice experiences [4, 9]. However, a rising in medical consumers’ right and unpredictable healthcare environments, clinical practice was limited to foster learner’s clinical reasoning competency [10]. To supplement it, high fidelity simulation (HFS) education is suggested as an education method [4, 11]. The degree of fidelity of simulator in healthcare education refers to similarity to reality, meaning the extent to which it is closer to reality ranging from low to high-fidelity [12]. A high-fidelity patient simulator is a full-body manikin that can be programmed to show a physical response to performance. HFS provides interaction between, the inputs of patient information and the environment, enabling interactive learning. The clinical situation can be reproduced in a sense of reality [13]. Simulation education is composed of scenario simulation and debriefing. Debriefing plays the role of connecting behavior and reflection. Debriefing confirms and reinforces the contents learned in lecture and makes the simulation experience a learning experience [11, 12]. Debriefing in simulation education is a learning strategy to improve reasoning competency [14, 15]. Through debriefing, in which the instructor and the learner review the clinical experiences, learners complete analyses and discussions, acquire knowledge, and improve critical thinking, communication skills, clinical performance ability, and clinical reasoning [14].

Dreifuerst [16] developed the Debriefing for Meaningful Learning (DML) method to foster nursing students’ clinical reasoning. According to DML, previous educational experience, reflection, knowledge and skills affect the students’ metacognition and nursing process application ability. These abilities cultivate the clinical reasoning. However, it was difficult to identify relationship due to complex connections among concepts in the process of clinical reasoning. In addition, previously reported debriefing methods were composed of brief frameworks such as environment, faculty and student role on debriefing stage [17, 18]. Much time and effort are required from the instructor for skillful debriefing. Cheng and colleagues study [19] reported that faculty training was very important to structure and qualified debriefing. Thus, it was needed to faculty guided debriefing for effective debriefing.

The aim of this study was to provide baseline data for the improvement of clinical reasoning of undergraduate students by developing a simulation education debriefing protocol that includes detailed processes, such as core questions for the instructor.

## Methods

The study was aimed to develop a simulation education debriefing protocol for undergraduate nursing students to foster their clinical reasoning.

### Research procedure

This study analyzed the data in compliance with the permission received from the Institutional Review Board (IRB No. 2015–0005-2) of the Nursing College of Yonsei University before the beginning of data collection. A preliminary debriefing protocol was developed using a comprehensive review of literatures. A debriefing protocol was established through completing verification of content validity using an expert group and additional in-depth interviews. The developed debriefing protocol was evaluated for applicability to undergraduate nursing students.

### Literature review

A comprehensive review of literatures was performed to develop a preliminary debriefing protocol. First, a literature search was performed on books and research journal articles on clinical reasoning and debriefing published from January 1980 to February 2016. A literature review was completed on research articles that developed or evaluated debriefing methods using database search such as PubMed, CINAHL, Riss, and KoreaMed. For literature search, MeSH terms “nursing education,” “patient simulation,” and “undergraduate” and non-MeSH terms “debriefing” and “clinical reasoning” were used as keywords. The inclusion criteria were studies with clinical reasoning and those studies conducted to develop debriefing methods. Research was limited to papers published in both English and Korean languages. We also excluded grey literature, such as editorial reports and peer reviewed articles. After searching corresponding literature lists and abstracts, full articles were collected and reviewed; manual searches in core journals were also included.

### Development of preliminary debriefing protocol

The preliminary debriefing protocol’s framework was organized by Fanning and Gaba’s [20] debriefing method, which has the structural elements, such as debriefer, simulation scenario experience, and debriefing steps. The framework consisted of description, analysis, and application steps. This method was commonly used in nursing and medicine HFS education. The debriefing contents, e.g., clinical reasoning attribution and core questions were established via review of the final selection of clinical reasoning and debriefing literatures. The core questions were arranged for the learning outcome. The analysis step learning outcome is that learners can analyze and reflect on their performances to solve patient’s health problems. The core questions corresponding to analysis attribution, i.e., ‘*what caused the patient’s health problem?*’, ‘*why do you think the health problem under discussion has the top priority?*’ were matched. Further educational contents such as video debriefing and nursing progress log can be obtained from previous studies [18, 21].

### Verification of validity

Content validity is defined as *“The degree to which an instrument has an appropriate sample of items for the construct being measured”* [22]*.* Content validity verification was completed by an expert group on core questions for each debriefing stage. A criterion for selecting experts was a faculty with simulation education experience of 2 years or more or a person experienced in simulation-related research. Verification of content validity was completed by 5 experts who satisfied the selection criteria. The validity of the debriefing protocol was verified via email using a scale from 1 point for “not appropriate at all” to 4 points for “very appropriate” for each question; opinions about items that needed to be modified or added were accepted. An index of content validation (CVI) was calculated and a question with 80% or more agreement was selected as a significant item [23].

### In-depth interview

Additional in-depth interviews were completed with 3 simulation education experts, using the developed debriefing protocol’s core questions. By rechecking the content validity and applicability, a detailed review of the debriefing protocol by experts was obtained. The in-depth interview questions were “*do you think that the clinical reasoning of healthcare students can be fostered through the debriefing protocol?*”; “*what are items that needed to be modified or added?*” Then, the debriefing protocol was modified and supplemented. A final debriefing protocol was developed through verification of content validity by an expert group and in-depth interviews.

### Applicability assessment

Using a modified debriefing protocol, the application assessment was accomplished. The focus group interview was used to examine the debriefing protocol effect. Kruger and Casey [24] suggested the sample size of 5 to 8 participants. In total, 8 students (2 groups of 4 students each) who were experienced in simulation education and clinical practice among senior students were originally recruited for application assessment. Then 7 students participated finally after one student withdrawing. For applicability assessment, 3 consecutive HFS were completed in 1-week intervals. After 3 HFS educations, focus group interviews were performed to examine the effect of debriefing protocol. The focus group interview questions consisted of open, introduction, transition, key, and ending questions [24]. The detailed questions were; ‘*were you able to connect with the clinical practice situation that you experiences earlier?*’; ‘*do you think clinical reasoning can be improved through the debriefing process?*’ The focus group interviews were conducted by an experienced qualitative interviewer.

## Results

In order to develop a debriefing protocol, a total of 12 articles including 5 studies related to clinical reasoning (e.q., Outcome Present State Test [OPT] model of clinical reasoning) and 7 studies related to debriefing (e.g., DML and GREAT simulation debriefing method) were used. Clinical reasoning competency guides healthcare providers to assess, understand, search, and classify information that influence the patient safety [13, 25]. It was classified into the attribution of perception, information processing, analysis, deliberation, metacognition, heuristics, intuition, inference, and logic [5]. The result of literature reviews of the included studies are summarized in Additional file 1.

The results of literature presented that more than half of the studies emphasized the 7 attributes of perception, information processing, analysis, consideration, metacognition, heuristics, and intuition. The attributes of heuristics and intuition, however, were excluded in this protocol. According to previous research [26], those attributions are used by expert level nurses who accumulated many clinical experiences. As a result, 5 clinical reasoning attributes, i.e. perception, information processing, analysis, deliberation, and metacognition were included.

### Extraction of core questions

The core questions derived from the literature reviews. Piaget [27] defined the perception as the thinking process of distinguish and judgment problems. The clinical reasoning studies [9, 28–30] emphasized that recognizing the patient priority problems were important for learners. In order to integrate these contents, the perception core questions such as ‘let’s discuss what health issues this patient has now’ and ‘what is the most important of the patient’s health problems we have so far discussed?’ were deduced.

Clustering related patient data and connecting the patient symptoms were significant to foster the information processing competency [9, 28–30]. On the basis of these researches, two information processing core questions were extracted.

Tanner [30] described that the analyzing attribute was resolved the simulation situation into its elements. Students noticed the reason of issue by subdividing the patients’ data [9, 29]. At the debriefing session, instructor provided the opportunity to analyze performance for being debriefed. Participants realized essential intervention and judgment [17, 20].

Deliberation was defined as the decision of alternatives by considering the best intervention to solve the problem [31]. Metacognition is a result of cognitive process. Through reflecting the learners’ cognition, they established the goals and plans [16, 18, 32]. Metacognition also included the evaluation of the learners’ thinking process [28, 30]. Analyzing deliberation and metacognition core questions were constructed from the analysis of previous clinical reasoning and debriefing researches.

### Development of debriefing protocol draft

The draft of debriefing protocol was composed of debriefing steps, learning outcomes, clinical reasoning attributions, core questions, and a faculty guide. Overall debriefing steps used the Fanning and Gaba’s [20] debriefing method. The core questions were arranged to pair the learning outcomes to be achieved at each stage using the debriefing steps of skills, analysis, and application.

The debriefing protocol added an instructor guide in order to reduce the time and effort for proficient debriefing. This protocol involved debriefing core questions from the literature review. In the faculty guide, reviewing the videos of simulation implementation was included in the description stage of debriefing. The video debriefing method had a positive effect of increasing the learners’ clinical reasoning, clinical judgment, and debriefing satisfaction via reflecting the simulation experience [21]. The reflection opportunity can bridge the gap between experiencing a simulation situation and understanding [20]. This reflection and experience are integrated into the learning.

In this study, reflective writing was included. Learners recognized the priority of the problems by analyzing objective/subjective data collected from patients through reflective writing. This process occurs in a self-directed manner, it can elicit learning motivation [18].

### Results of verification of validity

For validity of the debriefing protocol, content validity on the core questions of clinical reasoning was verified. The results showed that for all questions, the total CVI was 0.92; for each question, the CVI ranged from 0.80 to 1.00.

Among core questions to improve analytic competency “Why do you think the health problem under discussion has the top priority” was lower than those of other questions. The experts thought that the question duplicated a question in perception development: “What is the most important of the patient’s health problems we have so far discussed?”

The questions for perception attribute queried learners how they recognized the patients’ critical problems. On the other hand, the question for the analysis attribute asked reasons why they thought the most important issue among the patients’ health problems [30]. Therefore, both questions were used in this debriefing protocol.

### Results of in-depth interviews

Experts’ opinions presented that understanding the patients’ health problems by analyzing the contents, applying assess to evaluation processes and discussion at the debriefing stage is important for the improvement of clinical reasoning competency. When the protocol of this study is applied, its competency is expected to improve in simulation education.

Experts reported that “*the developed preliminary debriefing had many core questions. It looks like to take a long time to use in debriefing.*” Reflecting the experts’ opinion, the time to apply the debriefing protocol was measured. The experts expressed concerns that “*it might be difficult to apply to learners who did not have many opportunities to practice in similar simulation cases.*” By reflecting the previous steps, the core questions of metacognition attributes in the debriefing protocol were modified. The final debriefing protocol was consisted of debriefing stages, learning achievements, clinical reasoning attributes, core questions, and guideline for instructor (Additional file 2).

## Results of applicability evaluation

The senior level of undergraduate nursing students participated in the HFS education. Debriefing took about 1 h when applied the debriefing protocol. Two main topics were derived from 8 codes obtained from 16 condensed meanings by comparing, categorizing, and systematizing the analysis results of focus group interviews after debriefing.

The students reported getting an opportunity to analyze and organize the collected data to solve the health problems during the debriefing. They reported that “*we continued to connect data, and we felt more competencies in identifying the patients’ health problems*.” Through debriefing, learners had diverse experiences to recognize the priority health problem. Students promoted the cognition of health problems through connecting data and the related symptoms. Student also mentioned that “*I understood the flow of the reasoning process integrating the context of patient’s information*.” This opportunity was affirmative learning to students. Learners mentioned that their reasoning competency was improved and understood the overall flow of reasoning by applying the debriefing protocol. They stated that the reflection process of scenario helped to form a connection between the knowledge learned from lectures and the HFS experience.

The participants also reported that they experienced the self-directed learning environment through the debriefing protocol. Debriefer provided the enough time to present learner’s opinions and time to organize their deficiencies through immediate feedback. As well, students could have an interactive opportunity via the debriefing time. The detailed results of the debriefing protocol application are shown in Additional file 3.

## Discussion

This study developed a debriefing protocol to improve clinical reasoning of undergraduate students. Clinical reasoning is required as a core competency of healthcare providers. Simulation education is one of effective teaching method for fostering healthcare students’ competency [6, 37]. The most of previous studies of debriefing methods were not completed focusing clinical reasoning. Their usage as an education methods were limited to foster healthcare students’ clinical reasoning. Moreover, these were consist of brief frameworks, much time and effort are needed to the instructor for acquire debriefing skill. Therefore, the significance of this study analyzed the attributes of clinical reasoning that healthcare-related students could develop and improved them. For the instructor, it is possible to learn the debriefing efficiently.

The preliminary draft was modified following the results of validation using an expert group and in-depth interviews. The final established debriefing protocol was evaluated for applicability using both simulation education and focus group interviews with undergraduate nursing students.

The results and implications of the debriefing protocol were compared and interpreted with the previous studies. Existing the debriefing methods was composed of brief content. The instructors take much time and effort to complete skilled debriefing [33]. Recent review study [34] reported that most articles used not concrete method or vague structured debriefing. Only 9% reported that experienced debriefing instructor participation during the HFS. This debriefing protocol takes relatively less time for the instructor to acquaint the protocol and easy to use. The significance of this study is that faculty provides more than a certain level of debriefing to learners.

Debriefing is influenced by constructivism in theoretical framework. Constructivism was initiated by Piaget about 70 years ago, and his view of cognition is different from those of the previous theories. The most important aspect of this perspective is based on the learner’s experience, not the existing objectivity perspective, which sees knowledge as a description of the world [35]. Constructivist learning emphasizes contextual meaning of situation. It was the result of the learner’s understanding of errors that occur in the process of problem solving. Faculty didn’t intentionally reduce or avoid the students’ misconception [36]. Learning environments require the learner does not feel competition during the acquisition of knowledge. Within such environments, learners can express their own opinions in comfort, while instructors uncritically accept learners’ opinions, providing interactive feedback [13, 37]. Learning also arose in a diverse educational and communicational process [38]. However, mostly debriefings in simulation education are inefficient to learner-centered debriefing [39]. The purpose of simulation education is to apply the knowledge and integrate it with actual work rather than simple knowledge acquisition [11, 13]. Debriefing should provide an opportunity to reflect and analyze the completed nursing when the learner implemented scenario simulation individually. It should be an interactive feedback with the instructor.

In the in-depth interviews, experts expressed that debriefing protocol involved many core questions. It might be difficult to apply all of the developed questions to learners. The debriefing protocol’s feasibility, however, it took 1 hour. The HFS intervention time was 30 min. At the focus group interviews after the HFS, learners mentioned that they were able to talk fully about their experiences and opinions. Johnson-Russell and Bailey [40] stated that debriefing time is recommended 2 to 3 times more than the implementation time of the simulation. The time for the debriefing protocol appears to be within the applicable range.

The simulation learning experts advised that the application of debriefing protocol might be hard to apply to learners who did not have experiences with similar cases. The clinical sites are becoming more complicated; the patients’ diseases are consequently more diversified. It would be difficult to have the learners experience all the possible diseases from clinical practice. The advantage of HFS was to experience varies cases for learners; emergency, rarely clinical cases in controlled environment [16, 37]. It might be positive effect to undergraduate students’ problem recognition and interpretation competency. Considering these findings, the problem solving process competency might be difficult in short-term HFS. Continuing simulation education, the students’ clinical reasoning process should be improved even with a health problem that has not been experienced before.

In the focus group interviews, the learners expressed the cultivating clinical reasoning competencies such as perception, information processing and metacognition. The results indicated that they thought about assessments and interventions for solving patient’s health problems in connection with the knowledge. The students understood the flow of clinical reasoning process, which is an ability to solve patients’ health problems by repeated experiences of establishing care plans [11, 41]. Therefore, the debriefing protocol developed in this study should contribute to the improvement of undergraduate healthcare students’ clinical reasoning.

It was found that, in the applicability assessment of the debriefing protocol, the learners were provided with a self-directed learning environment. Arise from it, learning motivation was induced. Traditional debriefing environment was hierarchal feedback, it served as a barrier to foster learners’ motivation [37, 42]. The HFS environment using debriefing protocol was student perspective feedback and learner-centered debriefing. It was affected the learning motivation by having an opportunity to thoughtfully self-reflection, collaborative teamwork experience.

### Limitations

This study was conducted to establish the debriefing protocol for improving clinical reasoning for undergraduate healthcare students.

There were several limitations in this study. First, the validation of the results was limited because focus group interviews were performed after 3 HFS educations on 7 students. A quantitative study with a sufficient number of learners is needed to generalize the debriefing protocol.

Second, the evaluation of debriefing protocol effect did only nursing students. It is suggested to continuously evaluate the effects on the improvement of clinical reasoning by extensively applying the debriefing protocol to various levels and diversity healthcare majors of students.

## Conclusions

Clinical reasoning is an essential competency for healthcare providers. Simulation education is important to develop clinical reasoning for healthcare students. Debriefing is an essential for simulation education for achieve competency. This study was conducted to establish the debriefing protocol in order to improve of clinical reasoning. Through literature review, validation by experts and in-depth interviews, a final debriefing protocol was derived. It composed of debriefing stages, learning achievement, clinical reasoning attributes and core question and faculty guide. We expect that this study will cultivate undergraduate healthcare-related students’ clinical reasoning abilities. This study will offers to contribute patient safety.

## Additional files

Additional file 1: Clinical Reasoning Literature Review. (DOCX 21 kb)

Additional file 2: Debriefing Protocol. (DOCX 23 kb)

Additional file 3: Results of Debriefing Protocol Applicability Assessment. (DOCX 20 kb)

## Data Availability

Authors can confirm that all relevant data are included in the article and its supplementary information files.

## Abbreviations

AACN: American Association of Colleges of Nursing
DML: Debriefing for Meaningful Learning
HFS: High fidelity simulation
OPT: Outcome Present State Test
