# Supplementary material for: Development of simulation education debriefing protocol with faculty guide for enhancement clinical reasoning
Source: BMC Med Educ. 2019 Jun 11;19:197. doi: 10.1186/s12909-019-1633-8 (PMC6560888; doi:10.1186/s12909-019-1633-8)
Supplement: Supplementary file 1 — Clinical Reasoning Literature Review. (DOCX 21 kb) [file 12909_2019_1633_MOESM1_ESM.docx]

**Additional file 1 Clinical Reasoning Literature Review**

| **Author** | **Content** | **Clinical reasoning attribution** |
| --- | --- | --- |
| Kautz et al., (2005) | - Analyze logical clues on how patient problems interact and influence each other when many problems were considered at the same time - Priority of problems based on the analyzed content - Establish the care plan on the basis of priority - Conclude the clinical judgment through the perception on cognitive and metacognition process | Perception, Information process, Analysis, Metacognition, Inference, Logic |
| Tanner (2006) | - Through the heuristic reasoning process, healthcare provider noticed the initial clinical problems - Analyze the collected data - Connect knowledge and patient data via reasoning process - Understanding the difference between the collected data and expectation health patterns. - Based on previously similar clinical situations, healthcare providers understood the current clinical situation - Through a narrative reasoning process, healthcare providers comprehended an in-depth understanding of the context of patient’s situation - Evaluating the intervention was suitable for the evidence based practice | Perception, Information process, Analysis, Deliberation, Metacognition, Heuristics, Inference |
| Lasater (2007) | - Collect the objective and subjective data to solve the patient’s health problems - Analyze the collected data through the knowledge, experiences, and intuition - Understand the difference between the data collected information processing and expectation patterns. and understand priority of data - Perform nursing intervention by priority - Through a reflection process, learners’ realized their strength and weakness point - Leaners objectively evaluated if they could be improved | Perception, Information process, Analysis, Deliberation, Metacognition, Heuristics, Intuition |
| Benner et al., (2010). | - Understanding the priority of the clinical situation is the starting point of clinical reasoning - It is expressed as salience - Healthcare students established the priorities in nursing practice and deliberated their knowledge - With continued heuristic education, students acquired salience in familiar clinical environments - In order to foster a salience, instructor constructed educational strategies to understand contextual for learners | Perception, Information process, Analysis, Deliberation, Metacognition, Intuition |
