# Supplementary material for: Development of simulation education debriefing protocol with faculty guide for enhancement clinical reasoning
Source: BMC Med Educ. 2019 Jun 11;19:197. doi: 10.1186/s12909-019-1633-8 (PMC6560888; doi:10.1186/s12909-019-1633-8)
Supplement: Supplementary file 2 — Debriefing Protocol. (DOCX 23 kb) [file 12909_2019_1633_MOESM2_ESM.docx]

**Additional file 2 Debriefing Protocol**

| **Debriefing step** | **Learning outcome** | **Clinical reasoning attribution and core questions** | **Faculty guide** |
| --- | --- | --- | --- |
| Description | Learners can identify the problems occurred to patient | - Perception: Let's discuss what health issues this patient has now - Information process: What kinds of objective/subjective data have you gathered to understand the patient’s problem? | - Video-assisted debriefing - Provide the latest evidence-based guidelines or references - Prepare a patient assessf to evaluation process per team to the recognized patient’s health problem in the course of presenting the simulation |
| Analysis  Analysis | Learners s can analyze and reflect on their performances to solve patient’s health problems  Learners can analyze and reflect on their performances to solve patient’s health problems | - Analysis: What caused the patient’s health problem? - Information process: Let's connect the data gathered from the patient with the health problem - Perception: What is the most important of the patient’s health problems we have so far discussed? - Analysis: Why do you think the health problem under discussion has the top priority? - Deliberation: What kind of intervention have you provided to the patient, based on the priority of the patient’s problem? - Deliberation: What additional data should you have collected to understand the patient’s problem precisely and quickly? - Metacognition: What effect did you expect for the patient through the intervention you provided? - Metacognition: Which intervention have you not conducted to solve the patient’s health problem? | - Proceed with discussion in connection with the patient’s health problem, based on the patient assess to evaluation process. - Provide feedback on the objective/subjective data and interventions that the student has failed to recognize - Review and modify patient assess to evaluation based on faculty’s feedback |
| Application | Learners can review what they have learned and can establish an application plan | Metacognition: Let's think over what you did in the scenario simulation today, including good and poor things in your own performance?  Metacognition: Based on what you have learned today, make a plan on what kind of intervention you have to perform, if facing a patient like the scenario given today, when you are involved in a clinical practice or work as a healthcare provider. | - Apply to more patients with similar health problems to the simulation |
