# Supplementary material for: Development of simulation education debriefing protocol with faculty guide for enhancement clinical reasoning
Source: BMC Med Educ. 2019 Jun 11;19:197. doi: 10.1186/s12909-019-1633-8 (PMC6560888; doi:10.1186/s12909-019-1633-8)
Supplement: Supplementary file 3 — Results of Debriefing Protocol Applicability Assessment. (DOCX 20 kb) [file 12909_2019_1633_MOESM3_ESM.docx]

**Additional file 3 Results of Debriefing Protocol Application**

| **Themes** | **Code** | **Condensed meaning** |
| --- | --- | --- |
| Clinical reasoning  improvement | Perception | - Recognizing the priority health problem - Improvement in understanding the order of priority |
|  | Analysis | - Perceiving the importance of analyzing data from the patient |
|  | Information process | - Providing the opportunity to connect the collected data with the patient’s symptoms - Promoting the cognition of health problems through practicing connecting data and the related symptoms |
|  | Deliberation | - Comprehending the flow on the reasoning process that integrated the context of patient’s information - Realizing the overall flow for solving patient’s health problem from assessment to intervention |
|  | Metacognition | - Developing a link with the knowledge learned in class - Think about coping methods when similar clinical situation was faced through the application of patient assess to evaluation processes - Understanding the learners’ own deficiencies via reflecting their performances - An opportunity to think about additional possible interventions |
|  | Application competency | - Satisfaction from the application of cases to diverse patients |
| Self-directed  learning environment | Student-faculty  Interaction | - Satisfaction with interactive communication during debriefing - Providing the enough time to present learner’s opinions - Time to organize student’s deficiencies through immediate feedback |
